# Effect of Strain Rate on the Deformation Characteristic of AlN Ceramics under Scratching

**DOI:** 10.3390/mi12010077

**Published:** 2021-01-12

**Authors:** Shang Gao, Honggang Li, Renke Kang, Yu Zhang, Zhigang Dong

**Affiliations:** Key Laboratory for Precision and Non-Traditional Machining Technology of Ministry of Education, Dalian University of Technology, Dalian 116024, China; gaoshangf@gmail.com (S.G.); superhonggang@163.com (H.L.); Zhangyudlut@foxmail.com (Y.Z.); dongzg@dlut.edu.cn (Z.D.)

**Keywords:** aluminum nitride ceramics, nanoscratching, strain rate effect, stress field model, deformation characteristic

## Abstract

To clarify the influence mechanism of strain rate effect on deformation characteristics of aluminum nitride (AlN) ceramics, some varied-velocity nanoscratching tests were carried out using a Berkovich indenter in this paper. The deformation characteristics of the scratched grooves were observed using the scanning electron microscope. The experimental results showed higher scratch speed would lead to shallower penetration depth, fewer cracks, and indenter fewer slipping, which was more conducive to the plastic deformation of AlN ceramics. Considering the strain rate effect and the elastic recovery of material, a model for predicting the Berkovich indenter penetration depth under edge-forward mode was established. The prediction results were consistent with the experimental data, and the error was less than 5%, indicating that the model is effective. Based on the Boussinesq field, the Cerruti field, and the Sliding bubble field, a strain rate dependent scratch stress field model was established. The stress field revealed higher scratch speed may significantly reduce the maximum principal stress in the stress field under the indenter, which is the fundamental reason for reducing the crack damage and promoting the plastic deformation. The above study can provide theoretical guidance for reducing the processing damage of AlN ceramics.

## 1. Introduction

It is well known that aluminum nitride (AlN) ceramics have many excellent properties such as better thermal conductivity, reliable electrical insulation, nontoxicity, and thermal expansion coefficient matching with silicon [1,2,3]. Thus, AlN ceramics as heat dissipation substrates and packaging materials have been widely applied in microelectronics and semiconductors [4,5]. At present, hard abrasive machining is the most traditional and important processing method for AlN ceramic substrates, such as grinding [6] and lapping [7]. However, AlN ceramics belong to the hard and brittle materials, with high hardness and high brittleness. These traits often lead to a series of surface/subsurface damages during abrasive machining such as severe surface defects, brittle cracks, and residual stresses [8,9]. The processing damages of the AlN ceramics substrate will greatly affect the performance of semiconductor devices and seriously shorten the service life of devices [10]. To improve the strength and reliability of AlN ceramics substrates in semiconductor devices, the surface/subsurface damage caused by abrasive machining must be controlled and minimized. Grinding in the ductile region for AlN ceramics substrates is the most deal condition [11]. Therefore, it is very crucial to study the deformation characteristics and damage mechanism of AlN ceramics in the abrasive machining process, which is of great significance to control the damage, improve the surface quality, and guide the actual grinding process.

The scratch test can simulate the process of the single abrasive under scratching, which directly presents the detailed deformation characteristics of brittle materials. Malkin and Hwang [12] have found in the scratch tests on ceramics that two types of cracks were produced during the scratching process, medium/radial cracks and lateral cracks. The ductile removal can be achieved below the critical threshold load that does not produce medium/radial cracks. Cheng et al. [13] have studied the initiation of cracks in the glass during scratching and revealed that the critical threshold load for median crack can be estimated using the weak singularity in the deformation zone. Yang et al. [14] have investigated the effect of double-scratch interaction on cracks propagation during the scratching experiment of glass-ceramic with different scratch distances. In the range of scratch interaction, the larger scratching distance is more conducive to the ductile removal of glass-ceramic. Swab et al. [15] examined the effect of scratching loads on the equibiaxial flexure strength of soda-lime silicate and borosilicate glass by using the surface scratches method. After comparison, the soda-lime silicate glass appears to have less lateral cracking than borosilicate glass. In our previous study, Cai et al. [16] have carried out the variable force single-scratch and constant force double-scratch tests on AlN ceramics. The results showed that there is a brittle to plastic transition under cumulative loads. The increase in stress was the fundamental reason for the scratch direction change. Additionally, hard coating can greatly improve the cutting tool performance. The application of advanced coating materials is of great significance for improving the hardness, thermal stability, service life, and processing efficiency of cutting tools [17,18]. However, the wear of coating of cemented carbide tools is a major problem in engineering [19]. The scratch test is also an effective method to evaluate the adhesion strength and wear property of hard coating. Gonczy et al. [20] researched and wrote a new scratch adhesion test standard to instruct the scratch adhesion testing of thin, hard ceramic coating. Gong et al. [21] conducted the sliding wear testing on TiAlN and AlCrN coating based softer carbide substrates to evaluate the bonding property. Krzemien et al. [22] presented the micro-scratching technique to monitor stress changes caused by relaxation processes in multi-layer materials.

Meanwhile, some studies point out that the variation of strain rates caused by different abrasive scratching speeds has a great impact on material properties, such as strength and toughness [23,24]. The change of material properties will also affect the deformation characteristics and removal mechanism to a great extent [25,26]. A lot of researchers have studied hard and brittle materials with different scratch speeds and found that the strain rate effect of the material will significantly influence the scratch force, penetration depth, surface/subsurface deformation characteristics, and chip morphology in the nano-scratching process. Mukaiyama et al. [27] have studied the ductile-to-brittle transition of single-crystal silicon at different scratch speeds. The results presented that enhancing the scratch speed may lead to a decrease of transverse force during the ductile-to-brittle transition. Feng et al. [28] have conducted scratch tests with different scratch speeds on (0001) C-plane sapphire, revealing a higher proportion of plastic deformation with the increase in scratch speed. Yang et al. [29] have performed scratch tests on glass-ceramics using different speeds to explore the relationship between scratch speed and subsurface damage degree, indicating that increasing scratch speeds may inhibit the propagation of the median cracks and reduce the subsurface damage. Li et al. [30] have used a scanning electron microscope to analyze the effect of scratch speed on the scratch grooves and chips morphology of Gd_3_Ga_5_O_12_ (GGG) crystal, showing that higher scratch speed was beneficial to the formation of larger continuous chips. To sum up, the effect of strain rates induced by scratch speeds on deformation characteristics and damage mechanisms cannot be ignored. AlN ceramic is the polycrystalline material, which is liquid-phase sintered from numerous AlN grains with a few additives of yttrium oxide added to further improve the thermal conductivity and densification [31,32]. Its microstructure is completely different from other brittle materials such as silicon crystal and sapphire. At present, the strain rate effect has not been discussed in public reports on the deformation characteristics of the AlN ceramics. Therefore, it is necessary to clarify the effect of strain rate on the deformation characteristics of AlN ceramics.

In this study, we systematically studied the effect of strain rate on the deformation characteristics for AlN ceramic materials. The constant force single-scratch tests were performed with a standard Berkovich indenter at different scratch speeds. Scanning electron microscope (SEM) and laser scanning confocal microscope (LSCM) were used to observe the surface morphology, micro-damage, the penetration depth of scratch grooves, and the differences in deformation characteristics of AlN ceramics were compared. Finally, the influence mechanism of strain rate on the deformation characteristic was analyzed in detail, providing theoretical guidance for reducing the processing damage of AlN ceramics.

## 2. Materials and Methods

**Figure 1:**
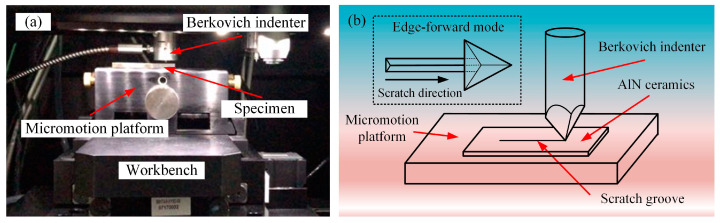
(**a**) G200 nanoindentation instrument (**b**) Outline of scratch tests.

The scratch tests with the scratch speeds of 0.1, 1, 10, and 50 μm/s were carried out, respectively, on the Nano Indenter G200 (Agilent, Santa Clara, CA, USA) with a standard Berkovich indenter (Figure 1a). The scratch direction of the Berkovich indenter is the edge-forward mode (Figure 1b). The normal force is a constant value of 15 mN, and the scratch length is 50 μm. To avoid the interaction between scratches, the distance between scratches is 100 μm. After scratch tests, the micromorphology of scratch grooves was observed by scanning electron microscope (SUPRA 55, Zeiss, Oberkochen, Germany). The cross-section profiles of scratch grooves were measured by laser scanning confocal microscope (VK-X200, KEYENCE, Osaka, Japan).

**Figure 2:**
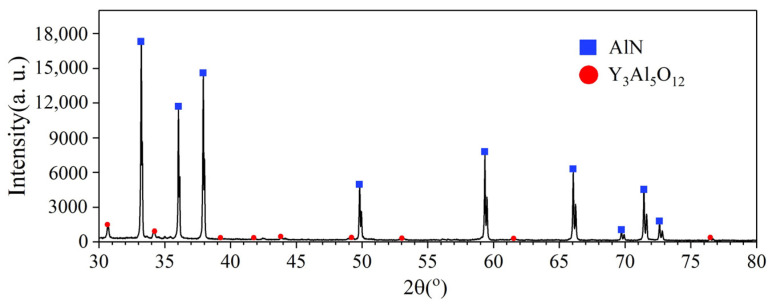
X-ray diffraction (XRD) spectrum of AlN ceramics.

The experimental samples were provided by Dongguan Kechenda Electronics Technology, Co., Ltd. The X-ray diffraction (XRD) spectrum shown in Figure 2 presents that the experimental sample contained some yttrium oxide impurities in addition to the main component of AlN with the wurtzite crystal structure. Table 1 shows the mechanical properties of this sample. Before the scratch tests, the 10 × 10 × 1 mm^3^ AlN ceramic sample was lapped with SiC loose abrasive, polished with 50 nm SiO_2_ slurry to the surface roughness *Ra* of 9 nm, and cleaned with deionized water and alcohol.

## 3. Results

### 3.1. Surface Morphologies of the Scratch Grooves

**Figure 3:**
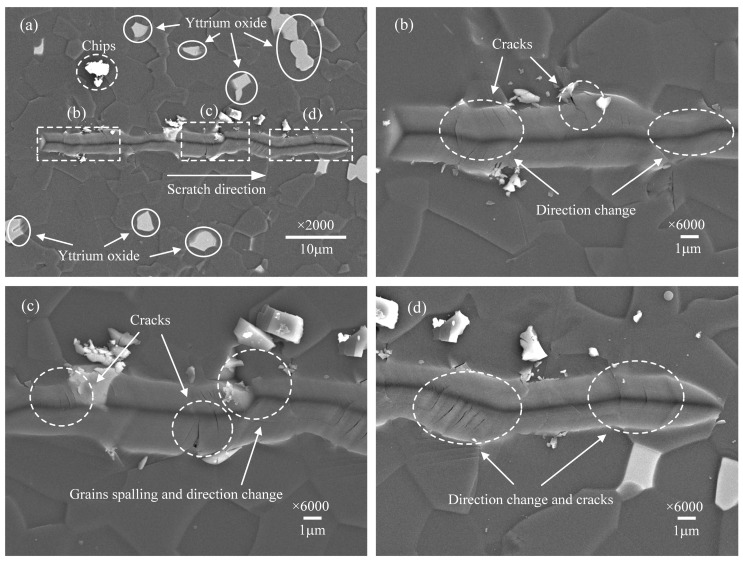
Surface morphology of the scratch groove at the scratch speed of 0.1 μm/s. (**a**) is the overall morphology. (**b**–**d**) are detailed enlarged views.

**Figure 4:**
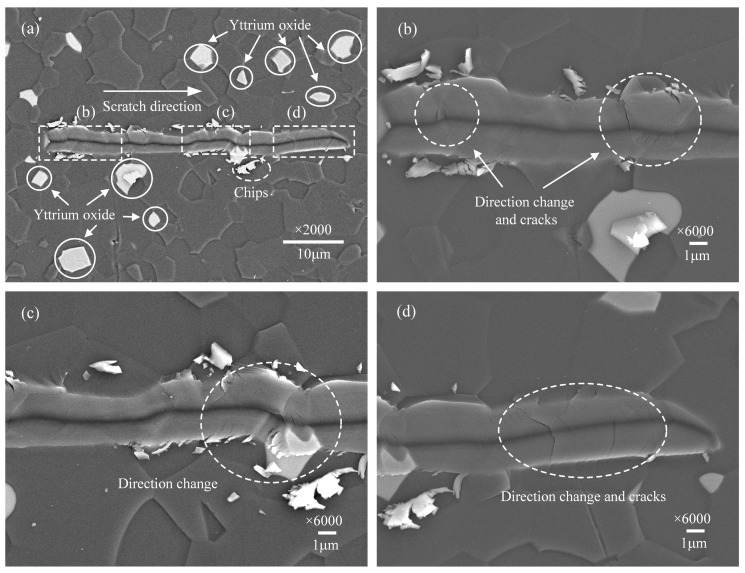
Surface morphology of the scratch groove at the scratch speed of 1 μm/s. (**a**) is the overall morphology. (**b**–**d**) are detailed enlarged views.

**Figure 5:**
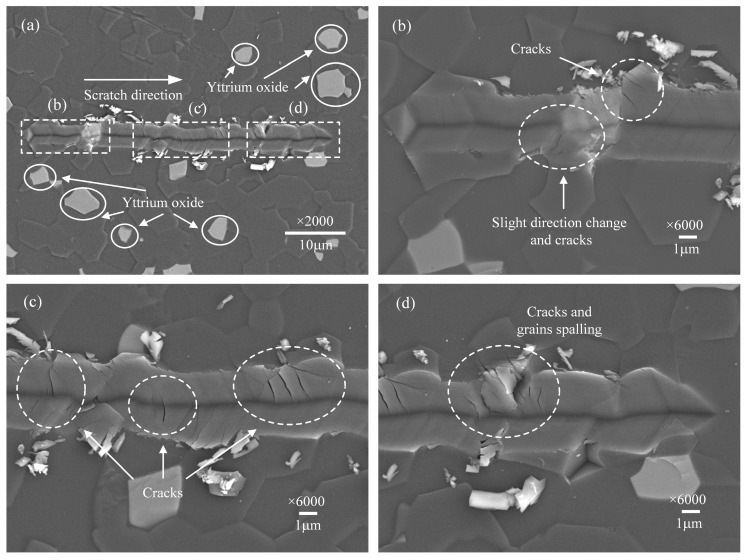
Surface morphology of the scratch groove at the scratch speed of 10 μm/s. (**a**) is the overall morphology. (**b**–**d**) are detailed enlarged views.

**Figure 6:**
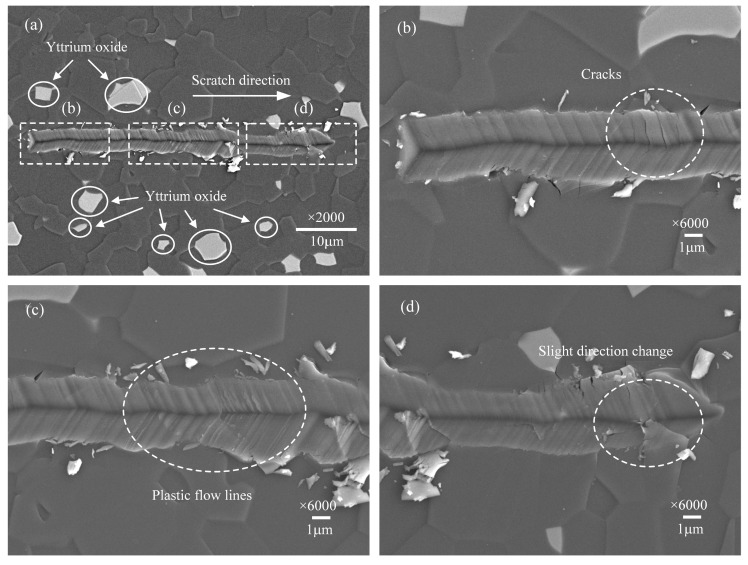
Surface morphology of the scratch groove at the scratch speed of 50 μm/s. (**a**) is the overall morphology. (**b**–**d**) are detailed enlarged views.

The surface morphologies of scratched grooves at scratch speeds of 0.1, 1, 10, and 50 μm/s are presented, respectively, in Figure 3, Figure 4, Figure 5 and Figure 6, observed by SEM. Figure 3a, Figure 4a, Figure 5a and Figure 6a show the overall morphologies of the scratched grooves, where the solid circular areas show a few white impurities of yttrium oxide. Figure 3b–d, Figure 4b–d, Figure 5b–d and Figure 6b–d are detailed enlarged views of the micro damage shown in the dashed boxed areas. Comparing these pictures, it is found that the scratch groove shown in Figure 3a has obvious tortuous features and the scratch direction has changed many times, which is not as straight as the single crystal material [30]. The scratch direction change indicates that the indenter has slipped. A large amount of micro-cracks appeared near the slippage of the indenter, as shown in Figure 3b,d. Grain spalling will occur when the cracks are severe, as shown in Figure 3c. It indicates that the slippage of the indenter has a great correlation with the cracks, and the material removal includes more than plastic deformation, but brittle fracture as well.

When the scratch speed further increased, the phenomenon of the scratch direction change in the scratch grooves gradually reduced, shown in Figure 4a and Figure 5a, but it can still be observed in Figure 4d and Figure 5c that micro-cracks propagate from the bottom to both sides of the scratch groove. There are also many micro-cracks at the grain boundary between AlN and yttrium oxide, as shown in Figure 5b. This demonstrates that even under a small load of 15 mN, micro-cracks are easily generated when AlN ceramics material is removed.

However, when the scratch speed increases to 50 μm/s, the morphology of the scratch groove shown in Figure 6a has changed prominently. The scratch groove morphology shown in Figure 6c,d has changed conspicuously, and some plastic flow streamlines exist that have never appeared before. Meanwhile, it can be seen from Figure 6a that slight changes in the scratch direction, indicating the indenter has no obvious slippage, and the micro-cracks are almost invisible, shown in Figure 6b. This illustrates that at a higher scratch speed, the plastic flow of the material can be enhanced, thereby inhibiting the indenter slip and the formation of micro-cracks.

### 3.2. The Maximum Slipping Distance and Penetration Depth of Indenter under Different Scratch Speeds

**Figure 7:**
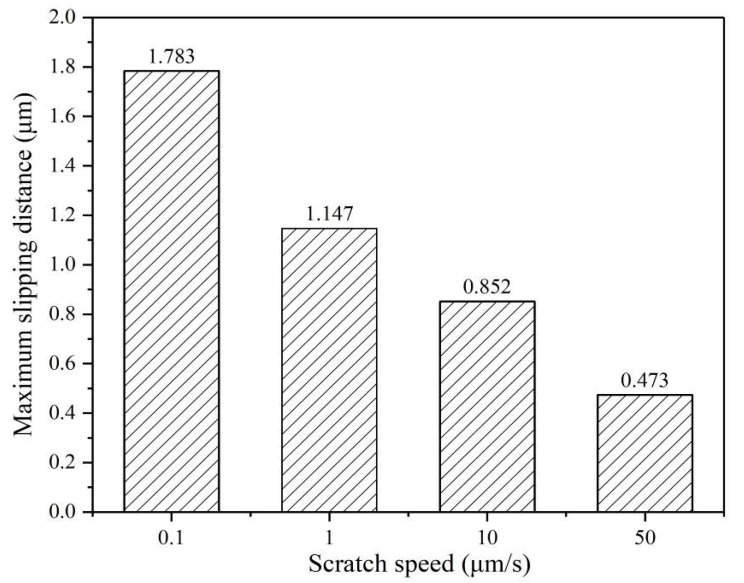
The maximum slipping distance of indenter perpendicular to the scratch direction at different speeds.

**Figure 8:**
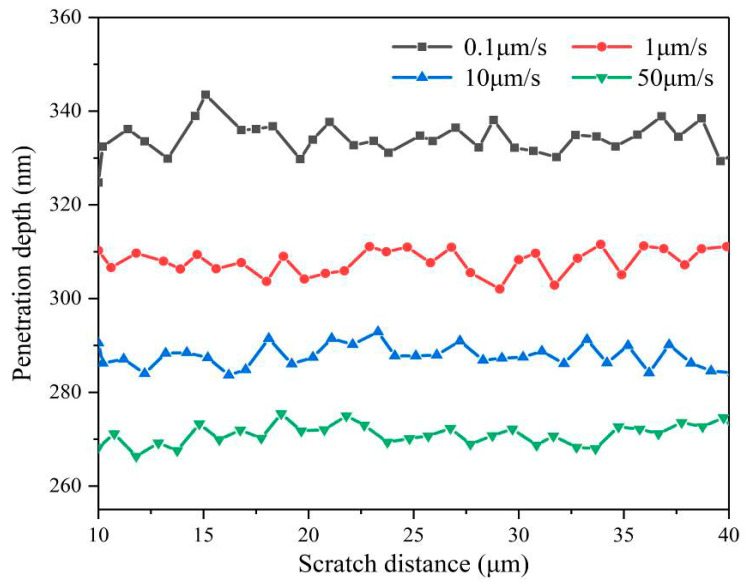
The penetration depth of the indenter at different scratch speeds.

Figure 7 shows the maximum slipping distance of the indenter perpendicular to the scratch direction under different scratch speeds. The maximum sliding distance refers to the maximum offset of the indenter relative to the straight scratch paths, which can be measured in the SEM image using the measuring scale. It is a relative value, which reflects the straightness of the scratch paths. Figure 8 shows the penetration depth of the indenter under different scratch speeds collected by the scratch test system. The scratch direction change is caused by indenter slippage. As the scratching speed increases, the slipping distances of the indenter are significantly decreased, and the penetration depths of the indenter are also slightly reduced, as shown in Figure 7 and Figure 8. The slipping distance of the indenter can implicitly reflect the crack damage. Therefore, these experimental phenomena indicate that higher scratch velocity and strain rate can contribute to the plastic flow of the AlN ceramics and effectively inhibit the indenter slipping and cracks damage.

## 4. Discussion

### 4.1. The Penetration Depth Prediction Model Considered with Strain Rate

The penetration depth of the indenter is an important factor in the scratching test for researching the deformation characteristics of materials [33]. Theoretically, the penetration depth under the constant force scratch tests should be a fixed value. However, the average penetration depth tends to decrease obviously with the scratch speed increases. Compared with the scratch speed at 0.1 μm/s, the penetration depth at a scratch speed of 50 μm/s is reduced by approximately 70 nm, as presented in Figure 8. This is an important reason for the plastic flow of AlN ceramics under higher scratch speed. It indicates that the strain rate has a certain relationship with the penetration depth, so it is necessary to establish a strain rate dependent penetration depth model of the Berkovich indenter. In the nanoscratching test, the scratch directions for Berkovich indenter include the edge-forward mode, the side-face forward mode, and the face-forward mode [34]. The existing model for predicting the penetration depth taking strain rate into account was only in the face-forward mode [30]. In our study, we adopted the edge forward mode, as shown in Figure 1, which greatly reduced the influence of the indenter spherical tip on the scratch direction change. Considering the strain rate effect and the elastic recovery of material, we discussed in detail the penetration depth in the edge-forward mode.

During the scratching process, the penetration depth of the indenter is mainly related to the normal force and the corresponding contact area between the indenter and workpiece. According to previous research [35], the contact area mainly depends on the yield stress of the material. In the subsequent studies [36,37], the dynamic average contact pressure *p_n_* is usually used to represent the stress of the material in the contact area. The relationship between normal force *F_n_*, dynamic average contact pressure *p_n_*, and the projection contact area *S* during scratching can be expressed by Equation (1) [34].

(1) $$F_{n}=p_{n}S$$

However, at different scratch speeds, the average contact pressure should also consider the strain rate effect [38,39]. The dynamic average contact pressure *p_n_* has a logarithmic relationship with the strain rate, as presented in Equation (2).

(2) $$p_{n}=p_{0}(m+k\ln(\dot{\varepsilon }))$$

where *m* and *k* are dimensionless coefficients. For hard and brittle materials, *m* and *k* can be taken as values 0.8 and 0.05, respectively. $\dot{\varepsilon }$ is the strain rate expressed by Equation (3) [40].

(3) $$\dot{\varepsilon }=\frac{v}{L}$$

where *v* is the scratch speed, *L* is the width of the scratch grooves measured by laser scanning confocal microscope, as is listed in Table 2.

Under the quasi-static state, the average contact stress *p*_0_ can be calculated by Equation (4), which is related to the material properties [41].

(4) $$p_{0}=\frac{2}{3}\frac{H}{2.8}(\frac{5}{3}+\ln(\frac{4(1-2\nu )\frac{H}{2.8}+E\tan\theta }{6(1-v)\frac{H}{2.8}}))$$

where *θ* is 19.7° for the Berkovich indenter, *H* is the hardness, *E* is the elastic modulus, and *ν* is the Poisson’s ratio. For AlN ceramic, these are listed in Table 1.

**Figure 9:**
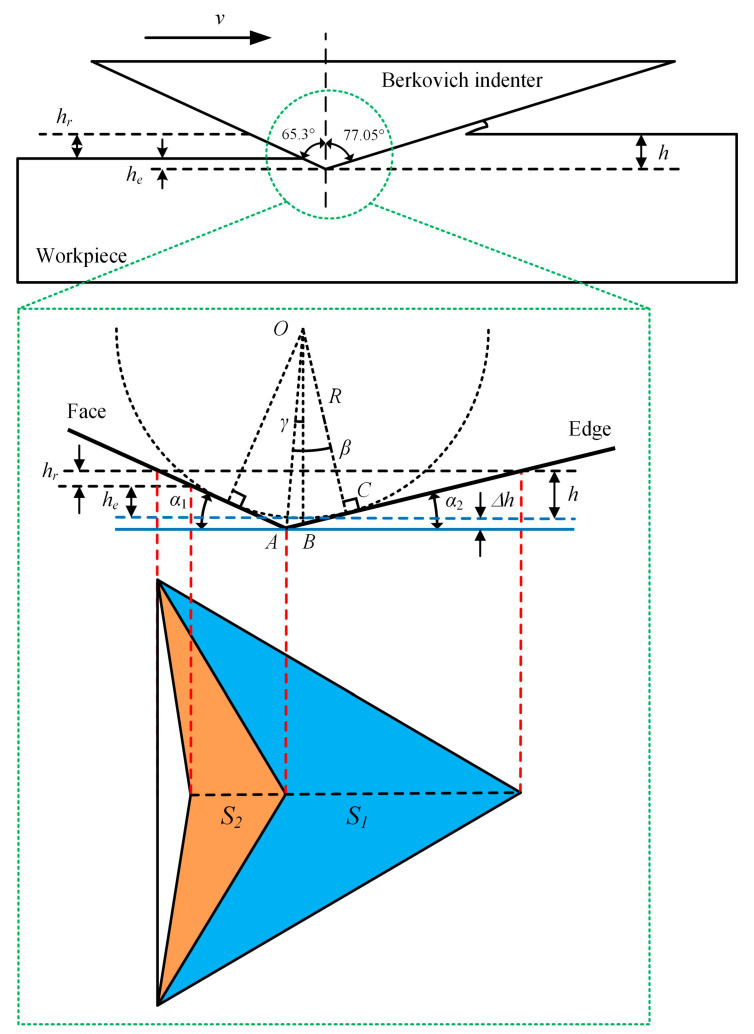
The projection contact area between the Berkovich indenter and the workpiece.

**Figure 10:**
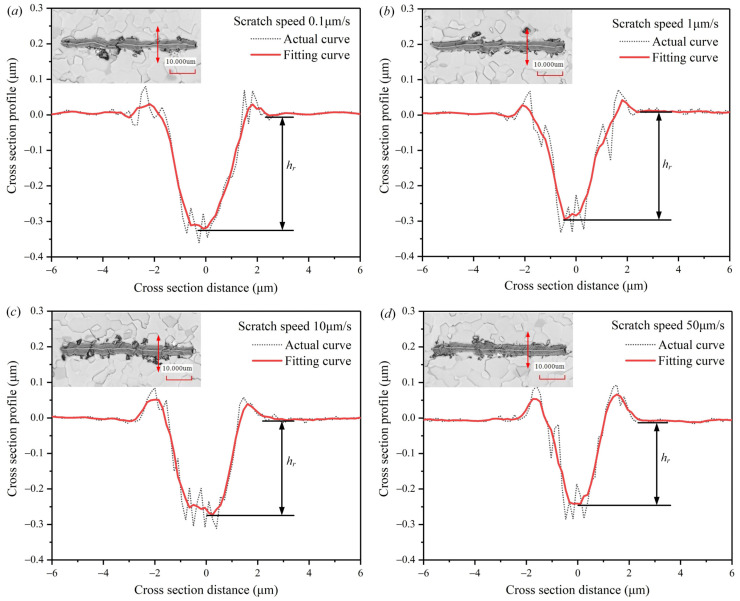
Cross-section profiles and residual depths of the scratches at different scratch speeds. (**a**) is the scratch speed at 0.1 μm/s. (**b**) is the scratch speed at 1 μm/s. (**c**) is the scratch speed at 10 μm/s. (**d**) is the scratch speed at 50 μm/s.

On the other hand, the penetration depth of the indenter is also related to the actual contact area between the indenter and workpiece during the scratching process. The real shape of a standard Berkovich indenter is a spherical crown with a radius of about 100 nm [30]. In this scratching test, the penetration depths are much larger than the radius of the Berkovich indenter. The elastic recovery of the material during the scratching process has a great influence on the actual contact area. It is assumed that the elastic recovery is completed immediately after the indenter scratching the workpiece surface. Figure 9 illustrates the geometric relationship between the actual contact area of the Berkovich indenter and the workpiece at edge-forward mode. The blue part *S_1_* is the scratch contact projected area. The yellow part *S_2_* is the elastic recovery contact projected area. *α_1_* = 24.7° and *α_2_* = 13° are the angles between the horizontal line with the face and edge of the Berkovich indenter, respectively. *R* about 100 nm is the radius of the indenter’s spherical crown. *h_r_* is the residual depth. *h_e_* is the depth of elastic recovery. *h* is the penetration depth, which is the sum of *h_e_* and *h_r_*. The residual depth *h_r_* can be measured by cross-section profiles of the scratches, which are collected by the laser scanning confocal microscope shown in Figure 10. The blue solid line is the theoretical scratched surface, and the blue dotted line is the actual scratched surface. Δ*h* is the difference between ideal depth and actual depth. *OA* is the distance from the center of the spherical crown to the theoretical vertex, and *OB* is the perpendicular distance from the center of the spherical crown to the theoretical scratched surface. The angle between *OA* and *OB* is *γ*, the angle between *OA* and *OC* is *β*.

According to the geometric relationship in Figure 9, the projection contact area *S* between the indenter and specimen can be deduced by Equations (5)–(7).

(5) $$S_{1}=2\sqrt{3}{\left(h+\Delta h\right)}^{2}\cot^{2}\alpha_{1}$$

(6) $$S_{2}=\sqrt{3}(h+\Delta h)(h+\Delta h-h_{r})\cot^{2}\alpha_{1}$$

(7) $$S=S_{1}+S_{2}=\sqrt{3}(h+\Delta h)\cot^{2}\alpha_{1}\left[3(h+\Delta h)-h_{r}\right]$$

where Δ*h* can be calculated by Equations (8)–(10).

(8) $$\gamma =\frac{\pi }{2}-\frac{1}{2}(\pi -\alpha_{1}-\alpha_{2})=\frac{1}{2}(\alpha_{1}-\alpha_{2})$$

(9) $$\beta =\alpha_{2}+\gamma =\frac{1}{2}\left(\alpha_{1}+\alpha_{2}\right)$$

(10) $$\Delta h=OB-R=\frac{\cos\gamma }{\cos\beta }R-R=R(\frac{\cos0.5(\alpha_{1}-\alpha_{2})}{\cos0.5(\alpha_{1}+\alpha_{2})}-1)$$

To sum up, the penetration depth prediction model of the Berkovich indenter at edge-forward mode can be deduced by Equations (1)–(10), as shown in Equation (11).

(11) $$h=\frac{h_{r}+\sqrt{h_{r}{}^{2}+4\sqrt{3}\frac{F_{n}}{p_{0}(m+k\ln(\frac{v}{L}))\cot^{2}\alpha_{1}}}}{6}-\Delta h$$

**Figure 11:**
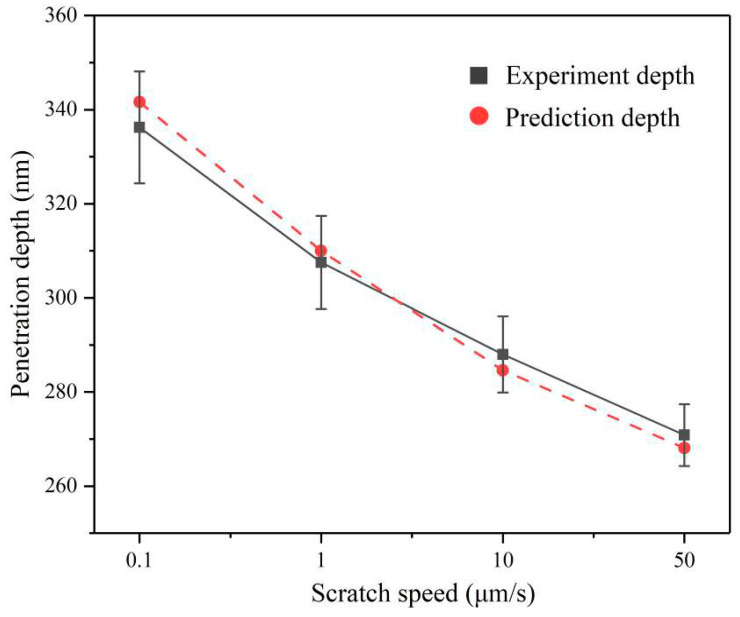
Prediction depths and experiment penetration depths at different scratch speeds.

The penetration depth *h* can be predicted by substituting the residual depth *h_r_* and the scratch speed *v* into Equation (11). Figure 11 shows the comparison of prediction and experiment penetration depth at different scratch speeds. The error between prediction and experiment data is less than 5%, indicating that the prediction penetration depth model of the Berkovich indenter for scratching AlN ceramics is effective.

Considering the strain rate effect, the increase in scratch speed will lead to the augment of the dynamic average contact pressure between the indenter and the AlN ceramic. Furthermore, under the normal force remaining constant, the reduction in the actual contact area will cause a decrease in the penetration depth of the Berkovich indenter. In the grinding process, the penetration depth refers to the depth of the cut of abrasives. Therefore, greatly increasing the scratch speed of abrasive will contribute to diminishing the depth of cut and reducing the processing damage, corresponding to the experimental phenomenon, which inspires the low-damage grinding AlN ceramic.

### 4.2. A Strain Rate Dependent Scratch Stress Field Model

**Figure 12:**
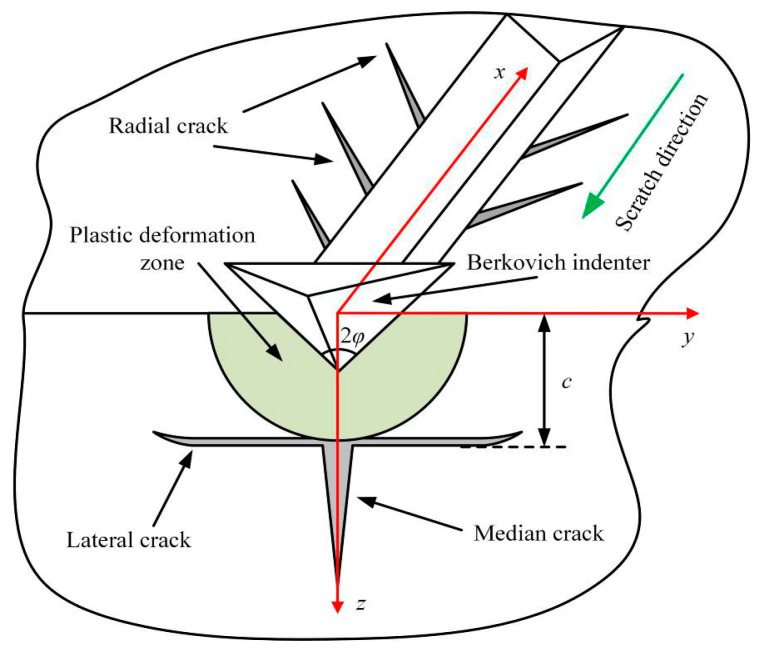
Diagram of the surface/subsurface damage during scratching.

According to previous research on hard and brittle materials, the lateral cracks and radial cracks will occur due to the stress [12,14], as shown in Figure 12. The AlN ceramics are a typically hard and brittle material. During the scratching process, the lateral cracks and radial cracks can be observed in the scratch groove. With the scratch speed gradually decreases, lateral cracks further expanding and interacting will cause severe indenter slip, as shown in Figure 3, Figure 4, Figure 5, Figure 6 and Figure 7. This indicates that the stress field under the scratch groove is the primary cause of cracks and indenter slip. Therefore, to examine the influence of strain rate effect on the deformation characteristics of AlN ceramics, it is extremely important to establish a strain rate dependent scratch stress field model.

During the scratching in brittle solids, the previous studies have proposed an analytical model for the stress field that regarded the elastic stress fields outside the plastic deformation zone as the superposition of the Boussinesq stress field *α_ij_*, the Cerruti stress field *β_ij_*, and the Sliding bubble stress field *γ_ij_* [42,43]. The stress field surrounding the scratch grooves can be expressed by Equation (12).

(12) $$\sigma_{ij}=k_{0}(\alpha_{ij}+k_{1}\beta_{ij})+k_{2}\gamma_{ij}$$

where subscripts *i* and *j* denote the directions of stress components such as *i*, *j* = *x*, *y*, and *z*. *k*_0_ represents the load state, which takes 1 when loading, and 0 when unloading. *k*_1_ represents the friction coefficient of the indenter obtained by experimental data, which is shown in Table 2. *k_2_=B/F_n_*, where *B* is the strength of the Sliding bubble field under per unit sliding length and *F_n_* is the normal force applied, and *k*_2_ can be calculated by Equation (13).

(13) $$k_{2}=\frac{B}{F_{n}}=f\frac{3\lambda^{2}}{4\pi^{2}(1-2\nu )(1+\nu )}\frac{E}{H}\cot\varphi$$

where *f* represents the compaction factor, and the value is 1 for the dense material [43]. *φ* = 65° represents the half-apex angle of Berkovich indenter. *H*, *E*, and *ν* represent the hardness, elastic modulus, and Poissonʼs ratio of the material, respectively. *λ* is a geometric parameter for the Berkovich indenter calculated by Equation (14) [44].

(14) $$\lambda =\sqrt{\pi /\sqrt{3}}$$

However, these stress field analysis models were only described under static conditions and ignored the effect of strain rate. For the hard and brittle materials, changes in strain rate can signally affect the micro-hardness of the material [45]. The dynamic hardness at different strain rates can be expressed by Equation (15).

(15) $$H=a+b\ln(\dot{\varepsilon })$$

where *a* is the static hardness of AlN ceramics listed in Table 1, and *b* = 0.3462 is the sensitivity of AlN ceramics hardness varying with strain rate [46].

Therefore, the strain rate dependent scratch stress field model can be deduced by Equations (12)–(15), as shown in Equation (16).

(16) $$\sigma_{ij}=k_{0}(\alpha_{ij}+k_{1}\beta_{ij})+\frac{3f\lambda^{2}E\cot\varphi }{4\pi^{2}(1-2\nu )(1+\nu )(a+b\ln(\dot{\varepsilon }))}\gamma_{ij}$$

According to the principles of fracture mechanics, when the maximum principal stress under the indenter exceeds the fracture strength of the material, radial and lateral cracks will follow. Lateral cracks further expand and interact during scratching, seriously affecting the deformation and removal of ceramics [14]. Hence, it is necessary to analyze the maximum principal stress at the place where lateral cracks occur.

The stress field which leads to the median cracks and lateral cracks is mainly distributed in the *y-c* plane, as shown in Figure 12. According to Equation (16), the *y*-component stress *σ_y_*, the *z*-component stress *σ_z_,* and the *yz*-component stress *σ_yz_* at the depth *z* = *c* in the *y*-*z* plane can be calculated as follows:

(17) $$\sigma_{yy}=k_{0}(\alpha_{yy}+k_{1}\beta_{yy})+\frac{3f\lambda^{2}E\cot\varphi }{4\pi^{2}(1-2\nu )(1+\nu )(a+b\ln(\dot{\varepsilon }))}\gamma_{yy}$$

(18) $$\sigma_{zz}=k_{0}(\alpha_{zz}+k_{1}\beta_{zz})+\frac{3f\lambda^{2}E\cot\varphi }{4\pi^{2}(1-2\nu )(1+\nu )(a+b\ln(\dot{\varepsilon }))}\gamma_{zz}$$

(19) $$\sigma_{yz}=k_{0}(\alpha_{yz}+k_{1}\beta_{yz})+\frac{3f\lambda^{2}E\cot\varphi }{4\pi^{2}(1-2\nu )(1+\nu )(a+b\ln(\dot{\varepsilon }))}\gamma_{yz}$$

The expressions for *α_yy_*, *α_zz_*, *α_yz_*, *β_yy_*, *β_zz_*, *β_yz_*, *γ_yy_*, *γ_zz_*, and *γ_yz_* at the Boussinesq field, the Cerruti field, and the Sliding bubble stress field are expressed as follows [43]:

(20) $$\alpha_{yy}(x,y,z)=\frac{F_{n}}{2\pi }\{\frac{1-2\nu }{r^{2}}[(1-\frac{z}{\rho })\frac{y^{2}-x^{2}}{r^{2}}+\frac{zx^{2}}{\rho^{3}}]-\frac{3zy^{2}}{\rho^{5}}\}$$

(21) $$\alpha_{zz}(x,y,z)=-\frac{3F_{n}}{2\pi }\frac{z^{3}}{\rho^{5}}$$

(22) $$\alpha_{yz}(x,y,z)=-\frac{3F_{n}}{2\pi }\frac{yz^{2}}{\rho^{5}}$$

(23) $$\beta_{yy}(x,y,z)=\frac{F_{n}}{2\pi }\{(1-2\nu )[\frac{x}{\rho^{3}}-\frac{x}{\rho {\left(\rho +z\right)}^{2}}+\frac{xy^{2}}{\rho^{3}{\left(\rho +z\right)}^{2}}+\frac{2xy^{2}}{\rho^{2}{\left(\rho +z\right)}^{3}}]-\frac{3xy^{2}}{\rho^{5}}\}$$

(24) $$\beta_{zz}(x,y,z)=-\frac{F_{n}}{2\pi }\frac{3xz^{2}}{\rho^{5}}$$

(25) $$\beta_{yz}(x,y,z)=-\frac{F_{n}}{2\pi }\frac{3xyz}{\rho^{5}}$$

(26) $$\begin{array}{l} \gamma_{yy}(x,y,z)=\frac{2F_{n}}{{\left(y^{2}+z^{2}\right)}^{3}}\{-2y^{2}(y^{2}-3z^{2})+\frac{x}{\rho^{5}}(2x^{4}y^{4}+6x^{2}y^{6}-2\nu x^{2}y^{6}+4y^{8}\\ -2\nu y^{8}-6x^{4}y^{2}z^{2}-7x^{2}y^{4}z^{2}-6\nu x^{2}y^{4}z^{2}-2y^{6}z^{2}-8\nu y^{6}z^{2}-12x^{2}y^{2}z^{4}-6\nu x^{2}y^{2}z^{4}\\ -15y^{4}z^{4}-12\nu y^{4}z^{4}+x^{2}z^{6}-2\nu x^{2}z^{6}-8y^{2}z^{6}-8\nu y^{2}z^{6}+z^{8}-2\nu z^{8})\} \end{array}$$

(27) $$\begin{array}{l} \gamma_{zz}(x,y,z)=\frac{2F_{n}^{}}{{\left(y^{2}+z^{2}\right)}^{3}}\{2z^{2}(z^{2}-3y^{2})+\frac{xz^{2}}{\rho^{5}}(6x^{4}y^{2}+15x^{2}y^{4}+9y^{6}\\ -2x^{4}z^{2}+10x^{2}y^{2}z^{2}+12y^{4}z^{2}-5x^{2}z^{4}-3y^{2}z^{4}-6z^{6})\} \end{array}$$

(28) $$\begin{array}{l} \gamma_{yz}(x,y,z)=2F_{n}\{-4yz\frac{\left(y^{2}-z^{2}\right)}{{\left(y^{2}+z^{2}\right)}^{3}}+(4x^{4}y^{2}+10x^{2}y^{4}+6y^{6}-4x^{4}z^{2}\\ +3y^{4}z^{2}-10x^{2}z^{4}-12y^{2}z^{4}-9z^{6})\frac{xyz}{{\left(y^{2}+z^{2}\right)}^{3}\rho^{5}}\} \end{array}$$

where $r^{2}=x^{2}+y^{2}$ and $\rho^{2}=x^{2}+y^{2}+z^{2}$.

According to Equations (17)–(28), the maximum principal stress *σ*_1_ at the depth *z* = *c* in the *y-z* plane can be calculated by Equation (29).

(29) $$\sigma_{1}=\frac{\sigma_{yy}+\sigma_{zz}}{2}+\sqrt{{\left(\frac{\sigma_{yy}-\sigma_{zz}}{2}\right)}^{2}+\sigma_{yz}{}^{2}}$$

**Figure 13:**
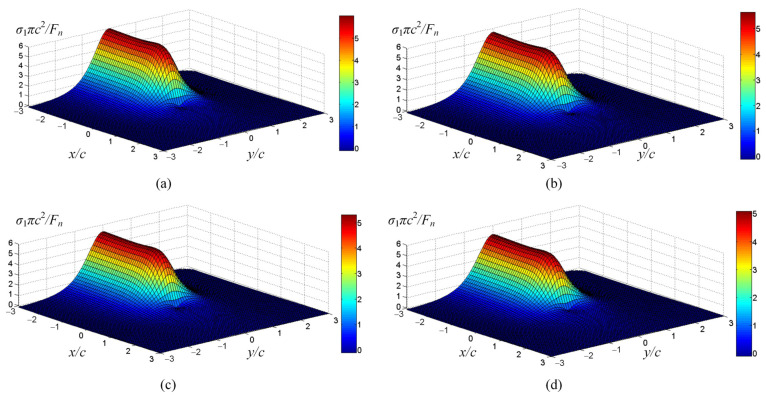
The principal stress distribution around the indenter at different scratch speeds. (**a**) is the scratch speed at 0.1 μm/s. (**b**) is the scratch speed at 1 μm/s. (**c**) is the scratch speed at 10 μm/s. (**d**) is the scratch speed at 50 μm/s.

**Figure 14:**
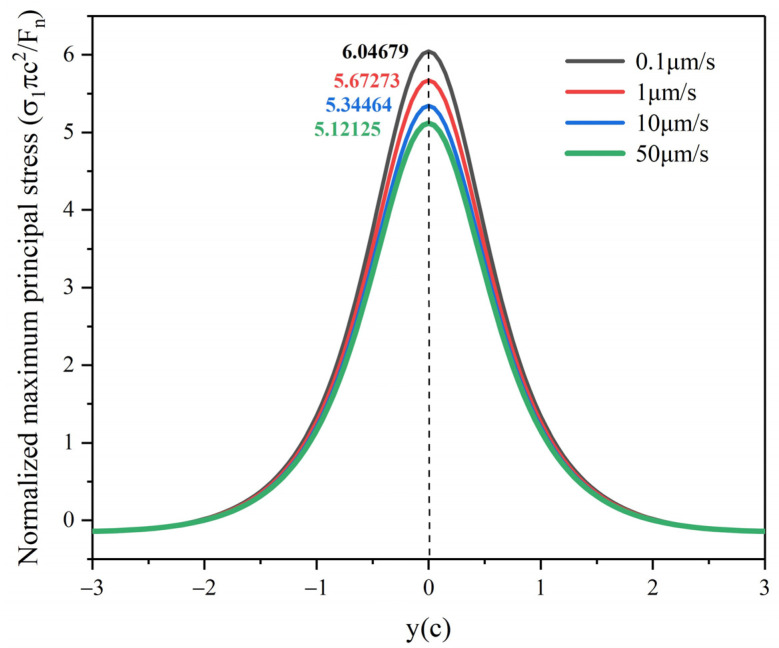
Normalized maximum principal stress at the depth z = c in the y–z plane under different scratch speeds.

To facilitate the comparison of the maximum principal stress at different scratch speeds, the maximum principal stress is normalized as *σ*_1_*πc*^2^*/F_n_*. According to the strain rate dependent scratch stress field model established in Equation (16), the principal stresses distribution around the indenter at scratch speeds of 0.1 μm/s, 1 μm/s, 10 μm/s, and 50 μm/s are shown in Figure 13. It is not difficult to find that the principal stress at the bottom of the indenter is the maximum during scratching, which will first lead to cracks in the area near the tip of the indenter, and then indenter slippage. Figure 14 shows that the normalized maximum principal stresses distribution below the indenter in the y–z plane at different scratch speeds, which is calculated by Equation (29). The extreme value of maximum principal stress can be used as a symbol of the possibility of cracks, which are located directly below the Berkovich indenter at y = 0, affecting the slipping distance of the indenter. With the scratch speed increases, the extreme value of maximum principal stress gradually decreases, which means that the cracks and indenter slipping distance are reduced, matching with the data in Figure 7.

The above comments indicate that the fundamental reason for the cracks and indenter slipping is that the maximum principal stress produced by scratching exceeds the fracture strength of AlN ceramics. Increasing the scratch speed can effectively reduce the maximum principal stress under the indenter, thus reducing cracks and indenter slipping. Therefore, in the grinding process, we can consider increasing the grinding speed to reduce surface damage caused by sharp abrasives. This paper offers an incentive referential significance for improving the surface quality of AlN ceramic processing.

## 5. Conclusions

The constant force single-scratch tests on AlN ceramics were carried out at different scratch speeds, and the following main conclusions are drawn:

(1) During the scratching process on AlN ceramics, the scratch groove was tortuous, and a large amount of cracks occur at the slippage of the Berkovich indenter, indicating that the deformation characteristics include plastic flowing and brittle fracture.
(2) Higher strain rates would result in shallower penetration depth, less cracks, and indenter fewer slipping. At a scratch speed of 50 μm/s, many distinct plastic streamlines appeared in the scratch groove, indicating that higher strain rates were beneficial to the plastic flow of AlN ceramics.
(3) A model for predicting the penetration depth of the Berkovich indenter under edge-forward mode was established, which takes into account the strain rate effect and the elastic recovery of material. The penetration depth model was consistent with the experimental results, and the error was less than 5%.
(4) Based on the Boussinesq stress field, Cerruti stress field, and the Sliding bubble stress field, a strain rate dependent scratch stress field model was established. The model analysis showed that enhancing the scratch speed may significantly reduce the maximum principal stress in the stress field under the indenter, which was the fundamental reason for reducing the cracks and indenter slipping.

## Figures and Tables

**Table 1:** Mechanical properties of AlN ceramics.

Parameters	Values
Poisson’s ratio *ν*	0.2
Elastic Modulus *E* (GPa)	366.4 ± 5
Hardness *H* (GPa)	15.21 ± 0.5

**Table 2:** Residual depth, groove width, and friction coefficient of scratches at different speeds

Parameters	Values			
Scratch speed *ν* (μm/s)	0.1	1	10	50
Residual depth *h_r_* (μm)	0.335	0.295	0.264	0.235
Groove width *L* (μm)	4.252	3.976	3.453	3.124
Friction coefficient	0.3087	0.3058	0.3041	0.2935
